# Thermodynamic aspects of ATP hydrolysis of actomyosin complex

**DOI:** 10.1007/s41048-016-0032-5

**Published:** 2016-12-22

**Authors:** Xuejun C. Zhang, Wei Feng

**Affiliations:** National Laboratory of Macromolecules, National Center of Protein Science-Beijing, Institute of Biophysics, Chinese Academy of Sciences, Beijing, 100101 China

## SUMMARY

Directional movement of cellular components is essential to all eukaryotic cells, and is driven by a mechanoenzymatic system consisting largely of the actin–myosin cytoskeletal protein complex. Structural and functional analysis of this complex has provided critical insights into the mechanisms that enforce and regulate the movement of intracellular components such as muscle fibers, vesicles, as well as organelles. However, the structural bases of energy coupling between ATP hydrolysis and force generation common for all myosins remain elusive. Here we briefly review the widely accepted concept of how the actin–myosin cycle functions. We then propose a model based on the assumption that most of the chemical energy stored in ATP is released in the step of ATP binding, not during the hydrolysis step per se. Importantly, we propose that this energy is used to dissociate myosin from the actin filament, the most energy-intensive step in the reaction of the actin–myosin functional cycle. This suggests that the dissociation step serves as the major energy storage, thus driving the remaining functional cycle of the actin–myosin complex.

## ACTOMYOSIN COMPLEX

**Fig. 1:**
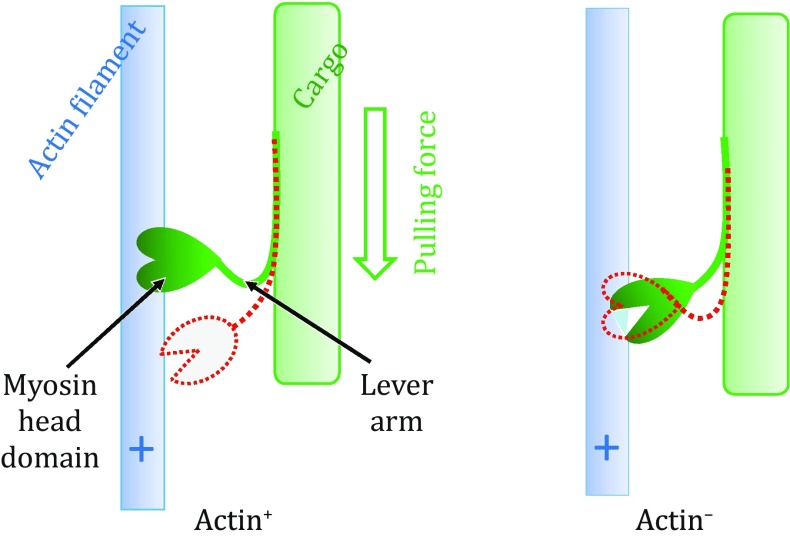
Schematic diagram of the actin–myosin functional cycle. Conformations in both actin^+^ and actin^−^ states are shown with solid objects. Subsequent conformations are indicated with *red dashed*-*line* models

Myosins belong to a large family of molecular motor proteins that harness the chemical energy released from ATP hydrolysis to generate unidirectional movement of cargo along actin filaments (F-actins) (Holmes 1997; Sweeney and Houdusse 2010; Geeves 2016). The complex formed by a myosin and an F-actin is referred to as actomyosin. Each functional cycle of actin–myosin interaction contains alternating steps of F-actin association and dissociation (Fig. 1) and is driven by hydrolysis of a single ATP molecule bound to myosin. Inside a living cell, the myosin is often physically associated with a cargo, for example, to the thick muscle filament in muscle contraction (Arakelian et al. 2015) or to the membrane vesicle in intracellular trafficking (Kruppa et al. 2016; Li et al. 2016). Multiple myosin proteins may attach to the same cargo simultaneously (Heissler and Sellers 2016a), and they may bind to multiple yet parallel F-actins, pulling the cargo to move along actin filaments. Such sliding movement of cellular components is observed in all eukaryotic cells, and because of their importance in a variety of cellular functions, actin and myosin are both present in multiple isotype forms.

Myosins contain a head domain (also called motor domain) that binds to ATP and F-actins alternatingly. This head domain is usually followed by a more-or-less rigid lever arm (also called a neck domain) and a cargo-targeting tail. Typically, the head domain is of pear-like shape with a molecular weight of ~90 kDa (Geeves 2016), and can be divided into two subdomains which are called upper and lower domains. The two subdomains are separated by a major cleft important for F-actin binding (Rayment et al. 1993). This cleft has two distinct states, namely a closed, actin-bound (actin^+^) state and an open, actin-free (actin^−^) state. They differ by a ~20° inter-subdomain rotation between the upper and lower domains (Holmes et al. 2003). An ATP-binding pocket is assembled mainly from structures of the upper domain, with minor contributions from the lower domain.

In the actin^+^ state, the ATP-binding pocket has an open conformation characterized by a low-affinity towards ATP. In contrast, in the actin^−^ state, the ATP-binding pocket adopts a high-affinity, closed conformation. It is estimated that ATP binding reduces the affinity between myosin and F-actin by four orders of magnitude (Geeves 2016). Thus, ATP and F-actin bind the head domain of myosin in a mutually exclusive manner. The communication mechanism between the ATP-binding pocket and actin-binding cleft has been elucidated structurally (Coureux et al. 2003; Ecken et al. 2016). Together, these findings have provided important insights into the molecular mechanisms for the extreme variations in affinity.

Depending on the presence or absence of ATP, the lever arm of myosin assumes two major conformations. In the actin^−^ state (ATP-bound), the lever arm assumes an “up” (backwards viewed from the head domain in Fig. 1) conformation, whereas in the actin^+^ state (ATP-free), the lever arm assumes a “down” (forward) conformation. This is true for all known myosin proteins moving towards the plus end of the actin filament. The only exception is myosin VI, which moves in the opposite direction (Sweeney and Houdusse 2010). Furthermore, the two conformations of the lever arm differ by a 70° rotation relative to the actin filament (Dominguez et al. 1998). This rotation generates a linear movement of 5–20 nm at the tip of the lever arm, depending on the length of the arm of myosin isoforms (Holmes 1997).

Currently, the hypothesis of the so-called “swinging lever arm” mechanism (Holmes 1997) is supported by a large amount of experimental data. According to this hypothesis, ATP binding to myosin causes dissociation of the head domain from F-actin. In turn, the unbound head domain diffuses towards the proceeding direction, directed by the swinging of the lever arm from its down conformation to the up conformation. The energy released during ATP hydrolysis is thought to be stored in subtle conformational changes inside the head domain; however, the nature of the changes remains unknown. In the next step of F-actin binding, the stored energy is released, causing the lever arm to switch (SW) from its up conformation back to the down one. Because the cargo is pulled by the lever arm against dragging force, this step is commonly called powerstroke.

It has been shown previously that the conformational transition of myosin associated with actin binding occurs in at least two stages. It starts from a weak interaction (with an initial contact area of ~1000 Å^2^ and *K*_d_ of sub-mmol/L) and ends with strong binding, ultimately resulting in isomerization (with an interface of ~2000 Å^2^ and *K*_d_ of ~10 nmol/L) (Siemankowski and White 1984; Holmes et al. 2003). A recent cryo-EM study showed that the myosin–actin interface within the actomyosin complex is dominated by hydrophobic interactions (Ecken et al. 2016). The four orders of magnitude difference in *K*_d_ between the two sub-states of weak and strong bindings indicates that the free energy of the myosin–actin binding is at least 10*RT* [i.e., *RT*ln(10^4^), where “*R*” is the universal gas constant and “*T*” is the absolute temperature]. In another work on muscle myosin, the equilibrium constant between the weak and strong actin–myosin binding modes was estimated to be ~280 (Coates et al. 1985), which suggests that a large (≥6*RT*) binding energy is released during the change of the binding modes.

One remaining question is how this free energy is first stored in the actin^−^ state, and then released during the powerstroke. Although it has been speculated that the driving force behind the powerstroke of myosin is the transition from a low-affinity myosin–actin complex to a high-affinity complex (Gigant et al. 2013), it is commonly assumed that the energy of powerstroke is stored in a spring-like apparatus (e.g., twist of a β-sheet) (Geeves 2016). Since there have been numerous excellent reviews on structural details of the actomyosin functional cycle (e.g., Holmes 1997; Sweeney and Houdusse 2010; Geeves 2016), we will instead focus on the thermodynamic aspects of the energy-coupling mechanism of actomyosin, and will argue against the spring-apparatus model.

## **TWO-STATE MODEL**

According to the swinging lever arm hypothesis, myosin must alternate between association and dissociation with the F-actin to fulfill its function. On the one hand, the strong binding between the myosin and F-actin indicates a large release of free energy, ∆*G*_L_(A)/*RT* ≪ 0 (where the letter “A” and subscript “L” stand for actin and its loading, respectively), during the F-actin loading. In the present work, we follow the convention that a thermodynamically favorable step is associated with a negative free-energy term. On the other hand, dissociation from the strong binding state [with a free-energy term ∆*G*_R_(A), i.e., −∆*G*_L_(A), where the subscript “R” stands for actin release] requires a large free-energy input, and general ATP hydrolysis is the only energy source for this dissociation process. Thereafter, we introduce the term “general ATP hydrolysis” to describe the entire multi-step ATP hydrolysis process, to distinguish this process from the specific, chemical reaction step of ATP hydrolysis (i.e., break of the O_βγ_–P_γ_ bond).

**Fig. 2:**
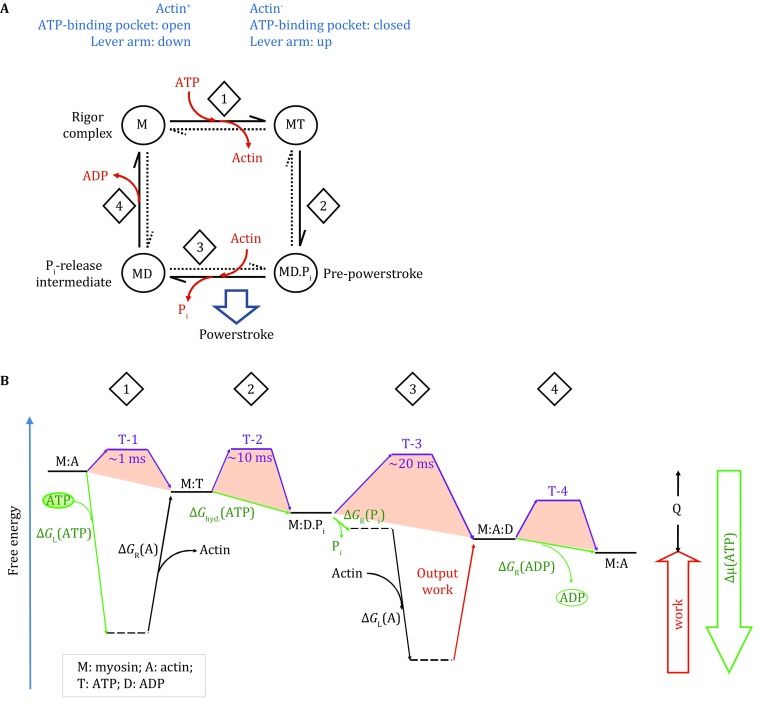
Two-state, four-step model of myosin. **A** King–Altman diagram of the actomyosin functional cycle. The two states are actin-bound (actin^+^) and actin-free (actin^−^) states. **B** Free-energy landscape of the actomyosin functional cycle. A free-energy landscape plot describes the thermodynamic relationship between different states. *Horizontal lines* represent states. *Tilted lines* represent transitions between states. *Green arrows* are associated with the chemical potential of ATP. *Purple lines* are associated with transition state energy barriers. Subscripts “L” and “R” stand for energy terms associated with loading and releasing, respectively. Collectively, the steps shown in this plot must satisfy the first and second laws of thermodynamics. The starting and ending states are identical, only being differed by the dissipation of the Gibbs free energy (release of heat, *Q*) during one functional cycle. The estimated kinetic data at the transition states of myosin II were from Geeves (2016). Note that the transition rate changes exponentially with the change of the height of energy barrier, in accordance with Arrhenius theorem. *Notes* (i) many energy terms in the plot are variable, depending on the cellular/experimental conditions. For example, in case that the output work becomes zero (i.e., load free), the rate of *step*-*3* would increase significantly, because the backward transition becomes negligible. (ii) There are many ways for ∆*µ*(ATP) to become zero. For example, when [ADP] is very high, the transition state energy barrier of the *step*-*4* (*T*-*4*) becomes prohibitorily high, and the motor will virtually stop proceeding. (iii) In case that the bound “ATP” molecule is non-hydrolysable, the process will stop at *T*-*2*

Based on the observations of association and dissociation between actin and myosin, a simplified two-state model can be deduced for the actomyosin functional cycle. The two major states in this model are actin^+^ and actin^−^. Furthermore, one may add steps associated with ATP binding, hydrolysis, and product release. The resulting, two-state, four-step model can be represented by the King–Altman diagram (Fig. 2A). In the following discussion, the free-energy transduction theory is used to treat the actin–myosin system (Hill 1989). In particular, both myosin and F-actin are treated as a combined thermodynamic system, and their concentrations become irrelevant to the thermodynamic cycle to be discussed. In other words, the myosin molecule is considered to be attached with the F-actin molecule during the entire functional cycle, though of varied affinity. In such a combined system, the association and dissociation between myosin and F-actin are equivalent to different conformational states of one protein complex. Furthermore, a free-energy landscape plot (Fig. 2B) is a useful tool to visually represent the functional cycle of actomyosin, for instance, illustrating whether a given step is thermodynamically favorable (Zhang et al. 2015). While the vertical dimension of the landscape plot represents Gibbs free energy, the horizontal dimension can be considered as an alternative expression of the King–Altman plot. A similar, although less detailed, energy landscape plot has been introduced before (Hill and Eisenberg 1981). Since the thermodynamic difference between any two given states is independent of possible connecting paths, the two-state model accommodates more complicated mechanisms with more sub-steps, as long as the major thermodynamic cycle is maintained. Whether a specific part of this energy landscape is united or divided into sub-steps, the fundamental principle should remain unchanged. For example, the first step of the actomyosin functional cycle (Fig. 2) may include the formation of the ATP-binding pocket by multiple local conformational changes (Dominguez et al. 1998; Coureux et al. 2003). Similarly, the third step may include sub-steps of weak and strong binding between the myosin and F-actin as well as inorganic phosphate (P_i_) release (Holmes et al. 2003; Sweeney and Houdusse 2010).

Moreover, each step that is associated with a large conformational change may contain a local transition state which determines the kinetics of the given step (Greenberg et al. 2016). Such transition states (T-1–4) are presented schematically in Fig. 2B. In case of multiple sub-steps, the collective transition might be considered as a group of coupled steps rather than a single transition state in the classical sense. However, coupled sub-steps are by definition intertwined, and their sequential order often evades experimental determination. In these cases, only the on- and off-rates of collective transition are experimentally measurable. For certain myosins, the fourth step (i.e., ADP release) appears to be regulated by the powerstroke as well as cargo load (Batters and Veigel 2016). Since ADP dissociation from myosin is a prerequisite for both ATP loading and F-actin dissociation, the thermodynamic equilibrium of step-4 strongly influences the “duty ratio” of the actomyosin complex, i.e., the fraction of time when the myosin stays attached to the F-actin (Sweeney and Houdusse 2010). For myosins of high duty ratio, external forces (e.g., that from oligomerized and cooperative monomers and that from the cargo load) are able to change the transition state barrier of step-4 and to shift its equilibrium, thus affecting ADP release (Heissler and Sellers 2016a). In this sense, the step-4 performs function of a mechanosensor. This notion explains why dimerization of myosin drastically changes the dynamic property of actomyosin (Heissler and Sellers 2016b).

## **GENERAL ATP HYDROLYSIS**

The free energy released from ATP and GTP hydrolysis by virtue of nucleotide hydrolases drives most if not all (energy-consuming) biochemical reactions and processes in living cells. ATP hydrolysis is thought to provide free energy for subsequent reactions (as exemplified in the P-ATPase transporter), and GTP hydrolysis often reenergizes a reaction cycle by pulling the cycle out of a thermodynamic “dead end” (as exemplified in a variety of Ras-like small GTPases). Therefore, ATP is called universal energy currency for living cells, whereas GTPases are called molecular switches. However, a thermodynamics view does not require such a functional distinction, especially for cyclical processes. Structural homology between ATPases and GTPases provides bases for our understanding of their functional similarity. Both ATPase and GTPase belong to a large family of nucleotide hydrolases that contain the characteristic P-loop, switch-1 (SW1), and switch-2 (SW2) motifs for nucleotide binding and hydrolyses (Vetter and Wittinghofer 2001; Geeves 2016). Typically, binding of ATP and/or hydrolysis of ATP into ADP and P_i_ trigger conformational changes of SW1 and SW2, and such changes convert chemical energy into mechanical forces. The ATPase activity in myosin is one of many “exceptions” of the above two-category “rule,” and the free energy released from ATP hydrolysis is not directly used in the powerstroke.

ATP molecules are considered to contain high chemical energy, and their hydrolysis to ADP and P_i_ generates ~30 kJ/mol (~12*RT*) energy. While the mechanism of ATP hydrolysis has been studied extensively both experimentally and computationally (Kiani and Fischer 2016), the roles of ATP loading and release during the general ATP hydrolysis are often ignored. Since both ATP loading and product release are referred to the same intracellular pool, the change in Gibbs free energy of an ATP molecule during an actomyosin cycle is exactly the same as that by any other processes of general ATP hydrolysis in the cytoplasm. This released energy is defined as ATP chemical potential:

1 $$\Updelta \mu ({\text{ATP}})\mathop = \limits^{{\rm{def}}} RT\ln \left( {[{\text{ADP}}] \cdot \left[ {{\text{P}}_{\text{i}} } \right]/\left( {[{\text{ATP}}] \cdot K_{{{\text{eq}}.,{\text{W}}}} } \right)} \right),$$

where the subscript “W” indicates a reaction in water. Thus, Δ*μ*(ATP) is determined by the cellular contents (i.e., [ATP], [ADP], and [P_i_]), and it sets the upper limit of how much energy may be gained from consuming one mole of ATP (Hackney 2005). Under normal cellular conditions, the large negative value (approximately −12*RT*) of Δ*μ*(ATP) strongly favors general ATP hydrolysis. Furthermore, Δ*μ*(ATP) can be divided into three parts (Zhang et al. 2016): ATP loading, ∆*G*_L_(ATP); ATP hydrolysis, ∆*G*_hyd._(ATP); and product release, Δ*G*_R_(ADP·P_i_):

2 $$\Updelta G_{\text{L}} ({\text{ATP}})\mathop = \limits^{{\rm{def}}} RT\ln \left( {K_{\text{d}} ({\text{ATP}})/[{\text{ATP}}]} \right),$$

3 $$\Updelta G_{{{\text{hyd}} .}} ({\text{ATP}})\mathop = \limits^{{\rm{def}}} RT\ln \left( {\left( {K_{\text{d}} ({\text{ADP}}) \cdot K_{\text{d}} \left( {\text P_{{\text i}} } \right)} \right)/\left( {K_{\text{d}} ({\text{ATP}}) \cdot K_{{{\text{eq}}.,{\text{W}}}} } \right)} \right),$$

4 $$\Updelta G_{\text{R}} \left( {{\text{ADP}} \cdot {\text{P}}_{\text{i}} } \right)\mathop = \limits^{{\rm{def}}} RT\ln \left( {[{\text{ADP}}]/K_{\text{d}} ({\text{ADP}})} \right) + RT\ln \left( {\left[ {{\text{P}}_{\text{i}} } \right]/K_{\text{d}} \left( {{\text{P}}_{\text{i}} } \right)} \right).$$

It is noteworthy that ∆*G*_hyd._ is a property of the nucleotide triphosphatase, independent of either substrate or product concentrations.

In enzymology, it is a commonly accepted concept that an enzyme accelerates a chemical reaction by lowering the transition state barrier. This can be achieved by “borrowing” the enthalpy of the reaction to overcome the entropy penalty during orientating the substrate to a pro-reaction position. Thus, the free energy released from general ATP hydrolysis [i.e., ∆*µ*(ATP)] can be shuffled between the abovementioned three terms, depending on structural details of the ATPase (Hackney 2005). Therefore, in contrast to the widely accepted model, the step of ATP hydrolysis per se [i.e., Δ*G*_hyd._(ATP)] may not necessarily be the step that releases most of the free energy. In particular, because of the binding conformation, the O_βγ_–Pγ bond of the bound ATP molecule may no longer be considered as a high-energy bond. This energy redistribution would explain why ATP hydrolysis is not directly coupled with the powerstroke.

A significant portion of Δ*μ*(ATP) must be released during ATP loading of myosin, in order to be coupled with F-actin dissociation [Δ*G*_R_(A) > 0] (Fig. 2B). This ATP-binding energy [Δ*G*_L_(ATP) < 0] is so large that there is little of Δ*μ*(ATP) left for directly driving the powerstroke movement. Instead of being directly driven by Δ*G*_hyd._(ATP), the powerstroke will be powered by the binding energy of myosin with F-actin [∆*G*_L_(A) < 0], which is large compared with Δ*G*_hyd._(ATP) and Δ*G*_R_(ADP·P_i_). Thus, the binding between myosin and F-actin is most likely to be a major step of utilizing the ATP energy, in order for the actomyosin motor system to maintain high efficiency of energy conversion. In agreement with this argument, it has been shown previously that for a myosin ATPase (e.g., myosin II), the ATP binding is rapid and nearly irreversible (Holmes 1997), meaning that the corresponding *K*_d_(ATP) is often too small to be measured reliably. Furthermore, the cellular concentration of ATP is in the order of 1 mmol/L, and the *K*_d_ of ATP towards myosin is in the order of 10 nmol/L (Siemankowski and White 1984). As a consequence of such strong binding, the resulting energy term, ∆*G*_L_(ATP), is approx. −11*RT* [i.e., *−RT*ln(10^5^)]. In other words, most of the ATP chemical potential (>90% of the 12*RT* available) will be released in its loading step, which is comparable in amplitude with the abovementioned binding energy between actin and myosin. Since actin and ATP compete for the head domain of myosin (Holmes et al. 2003), the free energy of ATP loading, ∆*G*_L_(ATP), is most likely to be coupled with breaking of the strong actin–myosin binding. At physiological ATP concentration (~3 mmol/L in muscle), the dissociation rate constant of actomyosin complex is fast, being in the range of 2 × 10^3^–6 × 10^4^ s^−1^ (Siemankowski and White 1984). Such a high rate suggests that the actomyosin dissociation catalyzed by ATP loading is favorable both thermodynamically and kinetically. Furthermore, as mentioned above, any favorable ATP loading [∆*G*_L_(ATP) ≪ 0] occurs at the expense of reducing the hydrolysis power of bound ATP. Thus, the ATP hydrolysis step in the actomyosin complex is associated with a rather minor free-energy change [∆*G*_hyd._(ATP) ≈ 0], such that under certain conditions, the bound ADP and P_i_ may reform ATP in the actin^−^ state (Geeves 2016). Similarly, P_i_ release from myosin is a reversible process, as long as the lever arm is present in the “up” conformation (Sweeney and Houdusse 2010). In the absence of actin, the bound P_i_ has an average lifetime of ~20 s (Geeves 2016), indicating that P_i_ release per se is unlikely to generate a significant amount of energy for the powerstroke. In contrast, actin-binding-induced conformational change renders the step of P_i_ release irreversible (Sweeney and Houdusse 2010). In addition, the free energy associated with ADP release may be either negative (i.e., thermodynamically favorable), zero, or positive, depending on isoforms of myosin (Geeves 2016). Thus, the release of ADP is also unlikely to provide significant energy for the powerstroke, at least not as a universal mechanism for all myosins. In summary, the more powerful (more negative) Δ*G*_L_(ATP) is, the closer to zero Δ*G*_hyd._ and ∆*G*_R_(ADP·P_i_) will be. However, since ATP hydrolysis enables subsequent release of P_i_ followed by ADP, the step associated with Δ*G*_hyd._ is required for the sustainability of the functional cycle of myosin. For the myosin ATPase, an energetic portfolio of large negative Δ*G*_L_(ATP) and small ∆*G*_hyd._(ATP) was achieved by the evolution through reducing *K*_d_(ATP), as well as through forming the ATP-binding pocket very close to the transition state of ATP hydrolysis.

## **COMPARISON OF ATP HYDROLYSIS OF MYOSINS AND KINESINS**

**Fig. 3:**
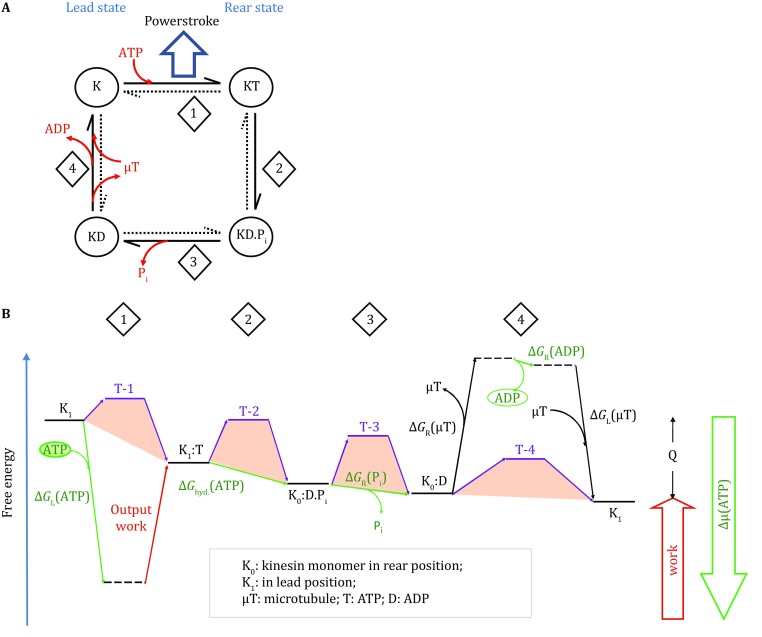
Two-state, four-step model of a kinesin protomer. **A** King–Altman diagram of the model of the kinesin–microtubule system. **B** Free-energy landscape of the functional cycle of a kinesin protomer

Kinesins form another major intracellular trafficking system, which moves cargoes unidirectionally along the microtubule track (Qian 1997; Wang et al. 2015). Like myosin, a kinesin protein also contains an ATP-dependent motor domain, a neck domain, and a cargo-targeting tail. Although there is little sequence homology between kinesins and myosins, their motor domains share some structural similarities in the ATP-binding region. Unlike myosin, kinesin proteins only function in a homodimer form, suggesting that coupling between the two protomers is essential for the functional cycle of kinesin. Structural and biochemical studies have shown that kinesin alternates between two major conformations, the lead- and rear-states, depending on the relative positions of the two protomers along the microtubule. The thermodynamic cycle of kinesin can be summarized with the diagrams shown in Fig. 3. Similar to myosin, kinesin releases most of the ATP chemical energy in the ATP-binding step, before ATP hydrolysis actually happens. However, unlike myosin, the energy released in the ATP-binding step of the “bound” promoter is immediately used in the powerstroke. In turn, this powerstroke moves the cargo in the proceeding direction, and at the same time facilitates the other (“moving”) protomer to overcome an energy barrier in its rear-to-lead transition (i.e., dissociation from the microtubule). In fact, the latter transition process is very fast, shorter than 2 ms. Once the moving protomer re-binds to the microtubule, the binding energy will be released to pay back the energy used for microtubule dissociation. For such coupling mechanism to work, kinesin must be present in its dimeric form. Therefore, kinesin uses a thermodynamic scheme similar in principle to that of myosin, with distinct differences related to the timing of the powerstroke in the ATP hydrolysis cycle.

## **CONCLUSION REMARKS**

Based on the above discussion and on the expansion of the widely accepted swinging lever arm hypothesis, we propose the following energy-coupling mechanism common to the functional cycle of various, if not all, actomyosin complexes. First, the tight ATP binding to actin^+^ myosin generates a considerable amount of free energy that is sufficient to break the actin–myosin interaction. Second, in the subsequent actin^−^ state, the lever arm of myosin is released from a high-energy (i.e., unstable in the absence of actin) “up” conformation, and the associated head domain is free to search for the next binding site on the F-actin in the proceeding direction, supported by a biased Brownian mechanism (Sweeney and Houdusse 2010). As observed in earlier studies, a heavy load slows down ADP release from actomyosin, thus increasing the duty ratio (Sweeney and Houdusse 2010). Furthermore, a heavy-load cargo restricts the search radius of the head domain to a more limited space, thus slowing down the speed of cargo movement. In contrast, a light-load cargo allows the head domain to search with longer step sizes and faster movement. It is in this step that ATP hydrolysis occurs. Third, once the head domain finds a proper binding site on the actin filament, it initiates weak binding which may further progress into a strong binding between the F-actin and the head domain of myosin. The energy released during the tight myosin–actin binding (which is made available by the previous ATP loading) not only induces ADP·P_i_ release, but also drives the conformational swinging of the lever arm, thus generating the powerstroke for the movement of the cargo. The main conceptual advance offered by our framework based on thermodynamic mechanisms described above is the following: it renders obsolete the requirement of the illusive intramolecular structural deformation commonly used to explain the high-capacity energy storage expected for driving the complete functional cycle of actomyosin. This newly proposed energy partition of ATP hydrolysis should enable the field of actin–myosin complexes to take into account thermodynamic considerations in future discussions of energy-coupling mechanisms.
